# History of Endometriosis Is Independently Associated with an Increased Risk of Ovarian Cancer

**DOI:** 10.3390/jpm12081337

**Published:** 2022-08-20

**Authors:** Antonio Sarría-Santamera, Zaukiya Khamitova, Arnur Gusmanov, Milan Terzic, Mar Polo-Santos, Miguel A. Ortega, Angel Asúnsolo

**Affiliations:** 1 Department of Medicine, Nazarbayev University School of Medicine, Nur-Sultan 020000, Kazakhstan; 2 National Research Center for Maternal and Child Health, Clinical Academic Department of Women’s Health, University Medical Center, Nur-Sultan 010000, Kazakhstan; 3 Department of Obstetrics, Gynecology and Reproductive Sciences, School of Medicine, University of Pittsburgh, Pittsburgh, PA 15213, USA; 4 Agency for Health Technology Assessment, Institute of Health Carlos III, 28029 Madrid, Spain; 5 Department of Medicine and Medical Specialities, Faculty of Medicine and Health Sciences, University of Alcalá, 28801 Alcalá de Henares, Spain; 6 Ramón y Cajal Institute of Sanitary Research (IRYCIS), 28034 Madrid, Spain; 7 Cancer Registry and Pathology Department, Hospital Universitario Principe de Asturias, 28805 Alcalá de Henares, Spain; 8 Department of Surgery, Medical and Social Sciences, Faculty of Medicine and Health Sciences, University of Alcala, 28801 Alcalá de Henares, Spain; 9 Department of Epidemiology and Biostatistics, Graduate School of Public Health and Health Policy, The City University of New York, New York, NY 10017, USA

**Keywords:** endometriosis, ovarian cancer, sociodemographic characteristics, risk

## Abstract

*Background:* Endometriosis is a complex gynecologic disorder that has been associated with a higher risk of ovarian cancer. The purpose of this work is to determine to what extent a history of endometriosis is a risk factor for ovarian cancer in a Spanish population. *Methods:* A retrospective case–control study was conducted using de-identified data from the Spanish National Health System’s “Primary Care Clinical Database” and “Hospital Minimum Basic Data Set” for the period 2013–2017. Multiple logistics regression analysis was conducted to determine associations between ovarian cancer and endometriosis controlled by sociodemographic characteristics and comorbidities. *Results:* Data from 608,980 women were analyzed, with 4505 presenting ovarian cancer. Endometriosis patients were shown to have a 2.66-fold increased risk of ovarian cancer when compared to those who did not have endometriosis by controlling age and other relevant comorbidities. *Conclusions:* This case–control study based on clinical administrative data has found that a history of endometriosis is independently associated with an increased risk of ovarian cancer. More research is needed to determine if a history of endometriosis affects survival results in ovarian cancer patients.

## 1. Introduction

Endometriosis is a benign condition, but it shares features with cancer, including metastatic-like behavior, tissue invasion, proliferation, angiogenesis, and decreased apoptosis. Epidemiologic studies have identified that women with endometriosis have an increased risk of ovarian cancer [1].

Endometriosis is a heterogeneous and complex disease, characterized by the presence of functionally active endometrial tissue, stroma, and glands outside the uterine cavity [2,3]. Numerous explanations have been proposed for its etiopathogenesis, such as retrograde menstrual bleeding, hormones, genetics, inflammation, or metaplastic transformation, but the exact mechanism of endometriotic lesions is still unknown [4].

The clinical presentation of endometriosis is highly variable [5]. Diagnosis of endometriosis is also challenging [6]. Probably due to diversity in the clinical course [3] and diagnostic complexities, estimates of the prevalence of endometriosis are quite variable [7], ranging from 1 to 5%, while incidence ranges between 1.4 and 3.5 per thousand per year, depending on the type of data and the design used for those analyses [8].

Existing therapies show significant variability in their effectiveness, possibly pointing to the fact that endometriosis may not be associated with a unique pathogenic process and that treatments should be optimized and personalized to specific sub-types of this disease [9]. Considering endometriosis as a pelvic gynecological disorder does not reflect its true scope and manifestations: endometriosis affects metabolism in liver and adipose tissue, leads to systemic inflammation, and alters gene expression in the brain that causes pain sensitization and mood disorders [10].

Endometriosis is often regarded as benign in nature, but some characteristics of malignant tumors (invasion, uncontrolled growth, etc.) are associated with this condition [11]. Ovarian cancer is one of the conditions that has been linked with endometriosis. Sampson described for the first time in 1925 an endometriosis-associated ovarian endometrioid carcinoma [12].

Ovarian cancer still shows low survival and is often diagnosed in the advanced stage given the late onset of symptoms and the lack of appropriate screening options [12]. Worldwide, there were 314,000 women diagnosed with ovarian cancer in 2020 and 207,000 ovarian cancer deaths, thereby ranking it eighth in terms of both cancer incidence and mortality among women globally [13]. As for Spain, based on data from the population-based registries, ovarian cancer may represent 3200–4000 new cases every year, most cases in women >45 years old [14].

Several factors may increase the risk for ovarian cancer, including older age; a family history of ovarian cancer; previous breast, uterine, or colorectal cancer; nulliparity; overweight; or obesity [15]. Ovarian cancer has also been linked to family cancer syndromes, some genetic mutations (BRCA1 and BRCA2), or women that have an Eastern European or Ashkenazi Jewish background. 

Ample evidence has linked endometriosis and ovarian cancer, but the relationship is not clear and is often controversial [16]. Several mechanisms have been suggested to explain the development of endometriosis-associated ovarian cancer (EAOC), including genetic alterations and hormonal and immunological factors [17]. Ovarian endometriosis is identified in about 30% of synchronous endometrial and ovarian cancers, especially of endometrioid type [18].

Both endometriosis and ovarian cancer are multifactorial diseases, so it is not surprising that more questions than answers remain. A recent systematic review and meta-analysis have quantified a positive association between endometriosis and ovarian cancer, estimating a 1.9-fold greater risk of ovarian cancer, with higher magnitudes and consistency of risk for clear cell (3.4-fold) and endometrioid (2.3-fold) histotypes of ovarian cancer [19].

No data have been published analyzing the possible association between endometriosis and ovarian cancer in Spain. In this sense, the aim of the present study is to investigate to what extent a medical history of endometriosis represents a risk factor for ovarian cancer development.

## 2. Materials and Methods

### 2.1. Study Design

A retrospective case–control study was undertaken by using de-identified data from 2 clinical administrative data sets: the “Primary Care Clinical Database” (PCCD) and the “Minimum Basic Data Set” (MBDS) of the Spanish National Health System.

### 2.2. Description of Databases

Controls were obtained from the PCCD, a large validated database being maintained by the Spanish Ministry of Health (SMoH). It includes de-identified data provided from Spanish Regional Health Services and obtained from their respective Primary Care Electronic Medical Record Systems. For this work, data of women aged 15–65 years from six Spanish regions, namely Andalusia, Basque Country, Cantabria, Catalonia, the Region of Murcia, and the Valencian Community, registered with their primary care centers in those regions from 2013 through 2017 were analyzed. The database consists of the following information from each patient: identification codes (IDs), income, labor situation, size of habitants, region, and country of origin as well as diagnoses and medicines prescribed by primary care doctors. IDs were anonymized by the Ministry of Health. The data for analysis do not have any variable that permits the identification of individual patients. For this work, comorbidities with a frequency of at least 5% were selected. Diagnoses were coded as a separate categorical variable that has “yes” and “no” subgroups. The diagnosis was coded using the International Classification of Primary Care, 2nd Edition. For the purpose of this study, a diagnosis of endometriosis was considered when a code of X99.01 was registered in the PCCD. Codes indicating a diagnosis of endometriosis are based on clinical provider reporting (primary care or hospital specialists) either in the PCCD or the MBDS. The data available do not permit to determine whether the diagnosis is based on signs and symptoms, diagnostic images, laparoscopic visualization, or biopsy.

Cases were obtained from the MBDS, also maintained by the SMoH. The MBDS contains anonymized data from all Spanish hospital discharges and is maintained by the SMoH with data provided by Regional Health Services, including sociodemographic and clinical data from all autonomous communities of Spain. The database has 64 variables that encompass all of the hospitalized patient’s information, such as financing regime, ID, residency, region, hospital care period, main diagnosis, secondary diagnoses, and performed procedures. Coding of the “main diagnosis”, 14 “secondary diagnoses”, and the “performed procedures” variables was conducted with the International Classification of Diseases 9th revision (ICD-9-CM). Inclusion criteria included cases with a code of ICD-9-CM 183.0. Endometriosis cases were those with code 617 in the diagnosis variables. The ICD-9-CM codes for other diagnoses are shown in Appendix A Table A1.

### 2.3. Databases Merging Strategy

For this study, the above-mentioned databases were merged into one combined database by identifying and matching the IDs and the variables’ names. However, before merging, cleaning of duplicates within the databases was performed based on the identification code (ID). If the identification codes of two or more patients were the same, their IDs were regarded as a pair of duplicate IDs. One of the duplicate IDs was kept while the other(s) were deleted from the database. The procedure for identifying, removing, and replacing duplicate IDs described above was repeated until no pairs of duplicates could be found in the database. Furthermore, if the information was absent in at least 2 variables, the patient was classified as having “missing information”. Patients with “missing information” were excluded from the database too.

As the two databases are differently structured, the corrections in the HDOCD made for easier matching of variables. It was restructured by transforming each of the ICD-9-CM coded diagnoses into a separate categorical variable (yes/no). To minimize the time spent on recodification, the list of diagnoses from the PCCD was taken as a reference to construct new variables in the HDOCD. The ICD-9-CM codes and corresponding diagnoses are listed in Table A1 in Appendix A. 

**Figure 1:**
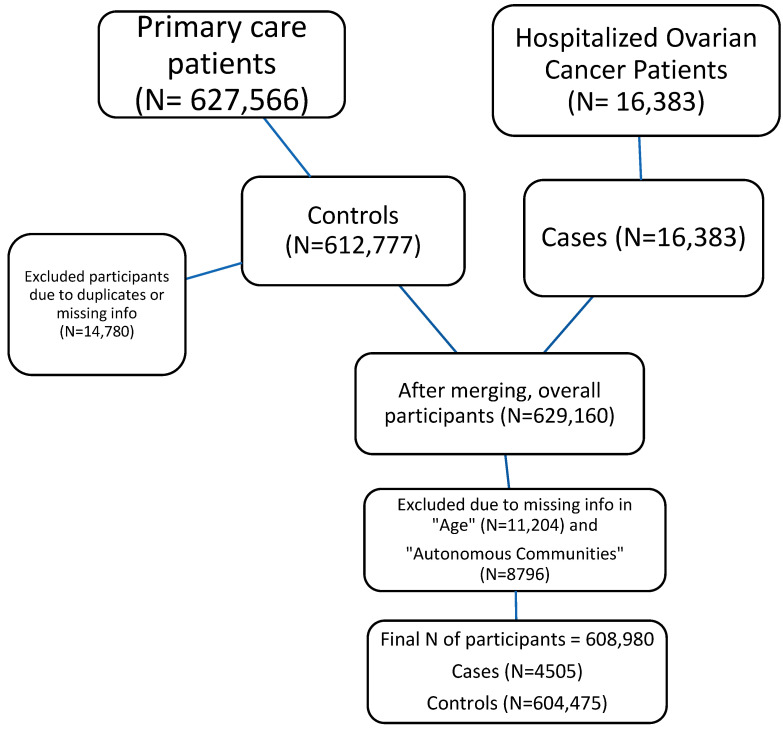
Illustrating the study design.

Afterward, we merged the two databases and repeated the procedure of removing duplicates. The combined database was divided into “cases” and “controls”. “Cases” were patients with diagnosed ovarian cancer, while “controls” were those patients without this diagnosis. Cases with inconsistencies in age, regions, or data before 2015 were eliminated. The flow chart of the study is presented in Figure 1.

### 2.4. Description of Variables

The final combined database included the main outcome (ovarian cancer) and 17 exposure variables, including sociodemographic characteristics such as age and residency region, and a list of comorbidities that were included because of their possible association with ovarian cancer or endometriosis (Annex ICD codes). The variable “Age” was divided into 3 categories (16–25, 26–45, and 46–65).

### 2.5. Data Analysis

The analysis included a univariate analysis of exposure to variables by finding their mean and standard deviation for numerical ones and by defining the proportions (%) for categorical ones. Bivariate analysis was performed by using two tests, namely the independent two-sample *t*-test and Pearson’s chi-square test, to determine the relationship between ovarian cancer (outcome variable) and exposure variables. The same analysis was performed to test the relationship between endometriosis (main predictor) and comorbidities. Moreover, an odds ratio (OR) was calculated for each obtained result. The ovarian cancer risk by endometriosis and other comorbidities was evaluated using unadjusted odds ratios (ORs), creating a comparative model using logistic regression analysis to show adjusted OR with 95% confidence interval (CI). The goodness of fit of the final model was checked to decide whether the model fits well enough. The obtained results are listed in Table 1, Table 2, Table 3, Table 4 and Table 5. All data analyses will be performed in STATA version 14.1 software (Stata Corp, College Station, TX, USA).

## 3. Results

Initially, two de-identified databases, the PCCD and the MBDS, included 627,566 “controls” and 16,383 “cases”. However, after checking for duplicative IDs, the MBDS contained none, while from the PCCD, 14,789 patients were excluded due to duplicated IDs or missing information.

After merging, the database contained 629,160 patients in total. On applying exclusion criteria to match cases and control subjects, 11,204 patients were dropped from the study due to outlying from the established age period of 16–65 years. Furthermore, the control individuals were matched with case patients from 2005 to 2015; therefore, the registered control subjects between 2015 and 2017 were eliminated from the database (N = 8976).

A total of 608,980 participants were included for the case–control study, of which 4505 were women with ovarian cancer (cases) and 604,475 were women without ovarian cancer (controls). The combined database consists of 18 variables, including sociodemographic information, ovarian cancer (outcome of interest), endometriosis (main predictor), and other comorbidities. The study population selection, including merging of databases and exclusion criteria, is shown in Figure 1.

### 3.1. Univariate Analysis

Descriptive characteristics of the study population are presented in Table 1. The mean age was 42 years (SD = 13.32). The age group from 46 to 65 years made up most of the participants (43.77%). Overall, 5247 (0.86%) women had endometriosis registered and 0.74% of the sample were diagnosed with ovarian cancer.

### 3.2. Bivariate Results: Ovarian Cancer

Detailed results for the association between ovarian cancer and age, endometriosis, or other diagnoses are presented in Table 2. The mean age of “cases” with ovarian cancer was 51 years, with 3347 (74.30%) of the patients being older adults, aged 46–65 years. The mean age of “controls” was 42 years, with an approximately equalized distribution of around 43% in adult groups (26–45; 46–65) (*p* < 0.05).

Endometriosis was identified in 2.22% of cases and 0.85% of controls (*p* < 0.05). In addition, four comorbidities showed a higher frequency in ovarian cancer cases: uterine fibromyoma was present in 4.28% of cases and 2.91% of controls (*p* < 0.05%), patients with ovarian cancer (10.63%) tended to have a twofold higher occurrence of allergy than those without it (5.30%) (*p* < 0.05), and gestational diabetes (2.91%) was reported more frequently among cases compared to controls (0.05%).

Other comorbidities had a lower prevalence among cases: migraine, obesity, psoriasis, hypothyroidism, and fibromyalgia (*p* < 0.05). 

### 3.3. Multivariate Results

Baseline characteristics were analyzed with bivariate analysis. The parameters that achieved a *p* < 0.05 were recruited for the multivariate analysis. Therefore, age, endometriosis, uterine fibromyoma, gestational diabetes, and allergy were recruited for the multivariate analysis. To check the independent effect of endometriosis, separate logistic models were created. Table 4 shows the adjusted OR of endometriosis in multivariate models including age and comorbidities found to be significantly associated with higher prevalence among cases. ORs for endometriosis were very similar after controlling for age and comorbidities shown to be also associated with ovarian cancer.

A final multiple logistic regression is presented in Table 5. All included variables were statistically significant.

After controlling for all other variables with a significant association, endometriosis showed to be independently associated to ovarian cancer. Risk of ovarian cancer also increased with age: the adjusted ORs of having ovarian cancer for women aged 26–45 and 46–65 were 3.74 [3.02–4.63] and 11.55 [9.39–14.21], respectively. History of uterine fibromyoma also increased the adjusted risk of ovarian cancer by 1.69 times [1.46–1.95]. Furthermore, ovarian cancer was shown to be significantly associated with a history of gestational diabetes (adjusted OR = 2.58 [1.79–3.27]) and allergy (adjusted OR = 2.11 [1.92–2.32]).

## 4. Discussion

The main finding of this work based on routinely collected data obtained from primary care records and from hospital discharge reports is the existence of an association between a history of endometriosis and ovarian cancer. This work has also identified a higher frequency of uterine fibromyoma, gestational diabetes, and allergy among women with ovarian cancer. Another relevant factor associated with a higher frequency of ovarian cancer is age. All those factors showed to be statistically independent factors not having an interaction effect with endometriosis. Conversely, this study has also found a series of conditions (migraine, obesity, psoriasis, hypothyroidism, and fibromyalgia) that may be associated with a lower prevalence in women with ovarian cancer.

Increasing evidence suggests that endometriosis patients are at higher risk of several chronic diseases [11]. Although the underlying mechanisms are not well understood, the available data suggest that endometriosis is not harmless with respect to women’s long-term health; these findings may have important implications in screening practices and in the management and care of endometriosis patients. In this sense, endometriosis has been linked to an increased risk of various cancer types [20]. In previous work conducted by Worley et al. [21], the association between endometriosis and ovarian cancer was clearly established. Endometriosis patients are typically younger, identified at an earlier stage, and have lower-grade ovarian cancer lesions [22]. Moreover, several retrospective studies in Japan have documented an increased risk of ovarian cancer in women with endometriosis [23]. For example, Kobayashi et al. studied the risk factors of cancer development in endometriosis [24]. They discovered that the incidence of endometriosis-related ovarian cancer was 0.72%, with a greater frequency corresponding with the patients’ rising age.

In our study, endometriosis was associated with a 2.66-fold greater risk of ovarian cancer compared to women without endometriosis. Ovarian cancer is known to develop in 0.3–1.6% of women with endometriosis, and endometriosis is observed in 4–29% of patients with ovarian cancer, implying a link between endometriosis and ovarian cancer [25]. This estimate is consistent with that reported by Burghaus et al. (2015) [26]. In their study, endometriosis was a relevant predictor for ovarian cancer in addition to other predictive factors (OR 2.63; 95% CI, 1.28 to 5.41). Aris et al. [27] found that the incidence of EAOC was 1.63% and the mean age was 48 years, 3 years younger than the age estimate obtained in our study. However, not all studies have reported an association between endometriosis and ovarian cancer. For example, Shen et al. [28] did not find any association between endometriosis and ovarian cancer. These findings support the association between endometriosis and ovarian cancer risk.

Ovarian cancer might not occur as a result of malignant transformation of glandular epithelial cells but might be caused by eutopic endometrial glandular epithelial cells with sufficient oncogenic mutations that are refluxed to engraft in the ovary [1]: those endometrial cysts may be considered to be “cancer from the beginning” [29].

The risk of developing ovarian cancer increases with age. The risk of malignant transformation of endometriosis is also higher in women of older age [30].

On the other hand, we found that uterine fibroids were also associated with a 1.69-fold increased risk of developing ovarian cancer. These data are opposed to the results obtained by some previous studies [28]. In one case–control study with a Chinese population, the adjusted odds ratio of women with a history of uterine fibroids developing ovarian cancer was 0.141 (95% CI: 0.085, 0.235; *p* < 0.0001). It is probable that the different results obtained in our study could be attributed to the sample size, as our study include a greater number of patients. In this line, Tseng et al. [31] also identified, in a broader sample size, that patients with uterine fibroids had a 2.26-fold higher risk of developing ovarian cancer than patients without uterine fibroids, and this risk could significantly decrease after myomectomy or hysterectomy. However, further studies are required before drawing relevant conclusions.

Gestational diabetes mellitus (GDM) constitutes one of the most representative and worrisome complications among pregnant women [31]. GDM is not only associated with adverse pregnancy outcomes but also has significant long-term implications on health conditions [32]. Previous studies have also reported it to be associated with increased risks of gynecologic cancers, although the evidence still yields inconsistent results [33,34,35]. Our study seems to indicate that patients with GDM display a 2.58-fold greater risk of suffering from ovarian cancer. In this line, Fuchs et al. [36] also reported that patients with a history of GDM had an increased risk for future breast, ovarian, and uterine malignancies.

Allergies are hypersensitivity reactions that occur through immunological mechanisms characterized by different soluble mediators, as well as specific cells of the immune system [37]. In recent decades, evidence has emerged relating this disease to cancer development. However, most of the results have been controversial and contradictory: while some evidence indicates that allergies can reduce the risk of cancer, other evidence indicates that it may increase this risk [38,39,40]. Currently, there are two major hypotheses explaining the positive/negative association between allergies and cancer. As allergies are characterized by immune hyper-responsiveness and enhanced immune surveillance, this could have prophylactic or anti-tumoral effects. Conversely, as allergies are mediated by specific T helper (Th)-2 responses, this may promote tumor growth, which may also benefit from the inflammatory environment [41]. Thus, it is difficult to establish the biological link between both conditions, and further studies are needed to deepen this relationship. In the case of ovarian cancer, compelling evidence suggests that there may be a negative or lack of association between allergies and ovarian cancer risk [42]. In our study, allergies appear to increase by 2.11-fold the risk of suffering from ovarian cancer. This could be due to the fact that endometriosis is more likely to occur in women with allergies, which could explain the increased risk of suffering from ovarian cancer. However, we cannot discard additional mechanisms working, as this field remains to be fully explored.

Overall, our study entails some limitations. The most important of them is the lack of information on how endometriosis has been diagnosed in controls, which may lead to an underdiagnosis of endometriosis in primary care patients. Endometriosis diagnosis was entirely dependent on codes obtained from primary care electronic medical records and hospital discharge reports, but it cannot be elucidated whether it was a clinical diagnosis or through ultrasound or laparoscopy with or without histological confirmation, established by the primary care provided or obtained after a referral to a specialist. Data on potential modifiers to the ICD-9 diagnosis, such as ‘suspected’, ‘ruled out’, ‘assured’, and ‘status post’, were not included as their use is inconsistent in this database. Some patients may have been diagnosed by a primary care doctor who documented endometriosis in a free-text section of the electronic medical record, without using the diagnosis codes, and such patients were not captured in the analysis. Other conditions such as irritable bowel syndrome, interstitial cystitis, chronic pelvic pain, and fibromyalgia may share symptoms with and could potentially be misdiagnosed as endometriosis [43].

Data on family history, gravity, parity, age of menarche, infertility, infections, lifestyle (diet, physical activity, and obesity), and potential environmental or genetic risk factors for ovarian cancer and endometriosis were not available [44].

Patients with ovarian cancer may be more intensively investigated, and the presence of a reported diagnosis of endometriosis in those patients may represent a more realistic estimation of the true prevalence among them. No specific data on the type of ovarian cancer are available. However, even for the cases, no specific data on the duration, treatments, clinical characteristics, location, stage, or extent of endometriosis were available. No information was available regarding the histological type of ovarian cancer in cases either.

Nevertheless, our work has certain important strengths to remark. The most important is the analysis of a large sample size, allowing us to examine the main effects as well as associations with potential comorbidities. The study sample contained a population of women from six autonomous communities of Spain and can thus be geographically diverse. In addition, we could assume there was a complete ascertainment of cases of ovarian cancer. Moreover, to the best of our knowledge, it is the first study that combined two differently structured databases from different sources to investigate the association between ovarian cancer and the history of endometriosis in women. The unique methodology of this study provided us a more complete and comprehensive understanding of the research problem.

## 5. Conclusions

Meta-analyses investigating the association between endometriosis and ovarian cancer typically report significant heterogeneity [19,45,46]. Likewise, the exact mechanisms of the endometriosis–ovarian cancer conversion are still not fully established, and the need for new approaches in the understanding of this connection is urgent [29,47].

This study conducted with a merged database including registries from hospitalizations and primary care has found a significant positive association between endometriosis and ovarian cancer. This association persists after adjusting for relevant variables such as age and comorbidities. Although these findings are similar to studies conducted in other settings and using other data, the characteristics of the data here analyzed and the possible underdiagnosis of endometriosis cases in primary care may weaken our conclusion. Future studies should be directed to determine whether the history of endometriosis disease influences survival outcomes among patients with different types of ovarian cancer. Furthermore, there is an urgent need to perform studies with epidemiologically related risk factors of ovarian cancer in Spain in order to prevent or facilitate an early diagnosis of this type of cancer, thus improving the survival and quality of life of the affected women.

## Figures and Tables

**Table 1:** Univariate analysis.

Variable Content	Total (N = 608,980)
Age, years	42.30 ± 13.32
Age categories	
16–25 year old	84,504 (13.88%)
26–45 year old	257,926 (42.35%)
46–65 year old	266,550 (43.77%)
Ovarian cancer	4505 (0.74%)
Endometriosis	5247 (0.86%)
Purpura	5524 (0.91%)
Uterine fibromyoma	15,411 (2.53%)
Irritable bowel syndrome (IBS)	9824 (1.61%)
Gestational diabetes	422 (0.07%)
Migraine	44,728 (7.34%)
Iron deficiency anemia	8430 (1.38%)
Obesity	34,103 (5.60%)
Psoriasis	12,200 (2.00%)
Hypothyroidism	59,969 (9.85%)
Fibromyalgia	17,279 (2.84%)
Allergy	32,535 (5.34%)
Ischemic heart disease	2359 (0.39%)
Essential hypertension	70,902 (11.64%)
Chronic venous hypertension	68,704 (11.28%)

**Table 2:** Bivariate analysis: the relationship between ovarian cancer and each of the other variables.

Total, N = 608,980	Ovarian Cancer Status		*p*-Value
Variable	No, N = 604,475	Yes, N = 4505	
Age (Mean ± SD)	42.23 ± 13.32	51.33 ± 10.06	<0.001 *
Age categories	N (%)	N (%)	
16–25	84,412 (13.96%)	92 (2.04%)	<0.001
26–45	256,860 (42.49%)	1066 (23.66%)	
46–65	263,203 (43.54%)	3347 (74.30%)	
Endometriosis	**5147 (0.85%)**	**100 (2.22%)**	**<0.001**
Purpura	5475 (0.91%)	49 (1.09%)	0.199
Uterine fibromyoma	**15,218 (2.52%)**	**193 (4.28%)**	**<0.001**
Irritable bowel syndrome	9745 (1.61%)	79 (1.75%)	0.453
Gestational diabetes	**291 (0.05%)**	**131 (2.91%)**	**<0.001**
Migraine	**44,705 (7.40%)**	**23 (0.51%)**	**<0.001**
Iron deficiency anemia	8359 (1.38%)	71 (1.58%)	0.269
Obesity	**33,963 (5.62%)**	**140 (3.11%)**	**<0.001**
Psoriasis	**12,185 (2.02%)**	**15 (0.33%)**	**<0.001**
Hypothyroidism	**59,793 (9.89%)**	**176 (3.91%)**	**<0.001**
Fibromyalgia	**17,247 (2.85%)**	**32 (0.71%)**	**<0.001**
Allergy	**32,056 (5.30%)**	**479 (10.63%)**	**<0.001**
Ischemic heart disease	2338 (0.39%)	21 (0.47%)	<0.001
Essential hypertension	70,352 (11.64%)	550 (12.21%)	0.235
Chronic venous hypertension	**68,661 (11.36%)**	**43 (0.95%)**	**<0.001**

* Independent two-sample *t*-test; Pearson’s chi-square test elsewhere.

**Table 3:** Bivariate analysis: the relationship between endometriosis and each of the other variables.

Total, N = 608,980	Endometriosis		*p*-Value
Variable	No, N = 603,733	Yes, N = 5247	
Age (Mean ± SD)	42.30 ± 13.36	43.32 ± 8.76	<0.001 *
Age categories	**N (%)**	**N (%)**	
16–25 year old	84,393 (13.98%)	111 (2.12%)	<0.001
26–45 year old	254,854 (42.21%)	3072 (58.55%)	
46–65 year old	264,486 (43.81%)	2064 (39.34%)	
Ovarian cancer	4405 (0.73%)	100 (1.91%)	<0.001
Purpura	5443 (0.90%)	81 (1.54%)	<0.001
Uterine fibromyoma	15,005 (2.49%)	406 (7.74%)	<0.001
Irritable bowel syndrome	9623 (1.59%)	201 (3.83%)	<0.001
Gestational diabetes	419 (0.07%)	3 (0.06%)	0.738 **
Migraine	44,127 (7.31%)	601 (11.45%)	<0.001
Iron deficiency anemia	8336 (1.38%)	94 (1.79%)	0.011
Obesity	33,831 (5.60%)	272 (5.18%)	0.188
Psoriasis	12,093 (2.00%)	107 (2.04%)	0.852
Hypothyroidism	59,390 (9.84%)	579 (11.03%)	0.004
Fibromyalgia	17,037 (2.82%)	242 (4.61%)	<0.001
Allergy	32,123 (5.32%)	412 (7.85%)	<0.001
Ischemic heart disease	2344 (0.39%)	15 (0.29%)	0.235
Essential hypertension	70,291 (11.64%)	611 (11.64%)	0.996
Chronic venous hypertension	68,112 (11.28%)	592 (11.28%)	0.998

* Independent two-sample *t*-test; ** Fischer’s exact test; Pearson’s chi-square test elsewhere.

**Table 4:** Odds Ratios of endometriosis interaction with other predictive variables.

Variable	OR	Adjusted OR
Endometriosis	2.64 [2.16; 3.23]	-
Endometriosis with Age	-	2.91 [2.38; 3.56]
Endometriosis with Age categories	-	2.66 [2.18; 3.25]
Endometriosis with Uterine fibromyoma	-	2.55 [2.09; 3.12]
Endometriosis with Gestational diabetes	-	2.68 [2.19; 3.27]
Endometriosis with Allergy	-	2.58 [2.11; 3.15]

**Table 5:** Multivariate logistic regression model.

Variable	OR	95% CI	Adjusted OR	95% CI
Age	1.06	[1.05; 1.06]	1.06	[1.05; 1.07]
16–25				
26–45	3.81	[3.08; 4.72]	3.74	[3.02; 4.63]
46–65	11.67	[9.48; 14.35]	11.55	[9.39; 14.21]
Uterine fibromyoma	1.73	[1.50; 2.00]	1.69	[1.46; 1.95]
Gestational diabetes	2.18	[1.49; 2.79]	2.58	[1.79; 3.27]
Allergy	2.12	[1.93; 2.34]	2.11	[1.92; 2.32]
Endometriosis	2.64	[2.16–3.23]	2.66	[2.17–3.26]

## Data Availability

The data used to support the findings of the present study are available from the corresponding author upon request.

## Appendix A

**Table A1:** ICD-9-CM codes for diagnoses mentioned in the study.

Diagnosis	ICD-9-CM
Cystitis	595.0–595.9 or 599
Uterine fibromyoma	218
Irritable bowel syndrome (IBS)	564
Gestational diabetes	V12
Acute stress	308
Eczema/dermatitis	692
Neurasthenia	300
Migraine	346
Iron deficiency anemia	280
Obesity	278.2 and 278
Psoriasis	696
Allergy	V14 or V15
Hypothyroidism	244
Fibromyalgia	729
Ovarian cancer	183.0
Ischemic heart disease	411 or 414
All forms of hypertension	401 or 405 or 459 or 642
Endometriosis	617.0
Purpura	287
Goiter	240.0 or 240.9
